# Identification of Cuproptosis-Related Subtypes in Lung Cancer, Characterization of Tumor Microenvironment Infiltration, and Establishment of a Prognostic Model

**DOI:** 10.1155/2022/7406636

**Published:** 2022-12-21

**Authors:** Jin Cui, Ying Xiong, Yuting Liu, Min Sun, Xinyue Gu, Luhui Zhong, Xiaohua Hong, Li Liu

**Affiliations:** Cancer Center, Union Hospital, Tongji Medical College, Huazhong University of Science and Technology, Wuhan, 430022 Hubei, China

## Abstract

Cuproptosis, a recently found kind of programmed cell death, has been linked to tumor development, prognosis, and therapeutic response. The roles of cuproptosis-related genes (CRG) in the tumor microenvironment (TME) are, nevertheless, unknown. We evaluated alterations in CRG and assessed the related expression patterns in 1445 lung cancer (LC) samples from three separate datasets, analyzing genetic, and transcriptional domains. We discovered two separate molecular subtypes of CRG and discovered that various subtypes of CRG were connected with patient clinical features and prognosis. Furthermore, we discovered connections between distinct CRG subtypes and TME cell infiltration features. The CRG_score was then developed and validated for predicting overall survival (OS). Following that, we investigated the relationship between CRG_score and the cancer stem cell (CSC) index and chemotherapeutic treatment sensitivity. In addition, we created a very accurate nomogram to increase the clinical usefulness of CRG_score. The potential roles of CRG in the tumor-immune-microenvironment, clinical characteristics, and prognosis in LC are demonstrated by our multiplex study. These findings expand our understanding of CRG in LC and may open up new options for assessing LC patients' prognosis and generating more effective immunotherapeutic treatments.

## 1. Introduction

Although lung cancer (LC) had been surpassed by breast cancer as the second most often diagnosed cancer in 2020, accounting for around 11.4 percent of all diagnosed cancers, it remained the leading cause of cancer fatalities, accounting for approximately 18.0 percent of all deaths. LC is expected to have 2.2 million new cancer diagnoses and 1.8 million deaths by 2020 [1]. The prognosis of LC is closely related to disease stage, with patients in stage IA having a 5-year survival rate of roughly 60%, whereas patients in stages II-IV have a 5-year survival rate ranging from 40% to fewer than 5%. Furthermore, patients with LC have a bad prognosis since more than 75% of them are in clinical stage III or IV at the time of diagnosis [2, 3]. Given the high morbidity and death rates as well as the poor prognosis of LC, the development of more effective prognostic and diagnostic models is critical. Copper is an essential trace element for the human body and is involved in a variety of biological processes in the human body [4]. According to recent research, copper levels in cancer patient's tumor tissue and serum are much greater than in healthy persons [5, 6]. Changes in intracellular copper levels have a significant impact on cancer start and progression [7]. Imbalances in copper homeostasis produced by genetic variations have been associated with potentially fatal disorders such as Wilson's disease [8, 9]. In addition, some researchers found that the reduction of serum copper concentration is related to the occurrence and development of endometrial cancer [10] and head and neck cancer [11]. Recently, researchers have discovered a novel cell death pathway called cuproptosis, and their studies have demonstrated that copper can directly bind to fatty acylated components of the tricarboxylic acid (TCA) cycle and cause toxic protein stress, ultimately leading to cell death [12–14]. Following the discovery of this essential and unique cell death mechanism, an increasing number of researchers are striving to find and uncover cuproptosis-related genes (CRG) implicated in copper-induced cell death.

The tumor microenvironment (TME) has been proven to have a significant impact on tumor genesis and development [15–17]. The complex relationship between tumor cells and nontumor cells in the TME influences cancer initiation and progression [18]. By producing cell signaling molecules, tumor cells can interact with surrounding cells via the circulatory and lymphatic systems, increase tumor angiogenesis, and drive immune cells to acquire immunological tolerance to tumor cells. Tumor-infiltrating immune cells (TIIC) within the TME have been proven in studies to predict cancer prognosis [19]. As a result, a complete study of CRG infiltration features in TME cells may give new possibilities for the underlying processes of LC as well as novel ways for predicting LC patients' response and prognosis to immunotherapy [20].

Using two computational algorithms, CIBERSORT and ESTIMATE, this study analyzed the expression patterns of CRG and provided a thorough overview of the immunological landscape within tumors. To begin, all LC samples were divided into two distinct subtypes based on CRG expression levels. Following that, LC patients were categorized into three genetic subgroups based on differentially expressed genes (DEG) discovered in the first two categories. In addition, we developed a scoring system to predict OS (OS) and describe LC immunological state to correctly predict patient prognosis and responsiveness to immunotherapy.

## 2. Materials and Methods

### 2.1. Data Sources

Data on gene expression (fragments per kilobase million, (FPKM)) and clinical and pathological features of LC patients were obtained from the gene expression omnibus (GEO) (https://www.ncbi.nlm.nih.gov/geo/) and the cancer genome atlas (TCGA) (https://portal.gdc.cancer.gov/) databases. Importantly, we incorporated patients' prognostic data in our collection. For the following studies, TCGA cohorts and two GEO cohorts (GSE68465 and GSE41271) were collected. We retrieved the raw “CELL” files and adjusted the background and quantile normalization. The FPKM values for TCGA-lung adenocarcinoma/lung squamous cell carcinoma (LUAD/LUSC) were translated to transcripts per kilobase million (TPM) and were thought to be identical to transcripts from the microarrays [21]. The three datasets used in this study were combined, and batch effects were removed using a “Combat” method. We eliminated patients with no or partial OS data. As a result, 1445 LC patients were selected for further study, using clinical data such as age, gender, T stage, N stage, follow-up time, and survival status.

### 2.2. Consensus Clustering Analysis of CRG

From earlier publications [4, 6–9, 22–25], nineteen CRG (NFE2L2, NLRP3, ATP7B, ATP7A, SLC31A1, FDX1, LIAS, LIPT1, LIPT2, DLD, DLAT, PDHA1, PDHB, MTF1, GLS, CDKN2A, DBT, GCSH, and DLST) were retrieved. To categorize patients into various molecular subtypes based on CRG expression, a consensus unsupervised clustering analysis was done using the R software package “ConsensusClusterPlus.” This clustering is done using the following criteria. To begin, the cumulative distribution function (CDF) curve gently and smoothly rises. Second, there was no group with small sample size. Finally, intergroup correlations increased while intergroup correlations declined following clustering. To study changes in CRG in biological processes, gene set variation analysis (GSVA) was done using the marker gene set (c2.cp.kegg.v7.2).

### 2.3. The Association of Molecular Subtypes with LC Clinical Characteristics and Prognosis

We investigated the association between genetic subtypes, clinical data, pathological characteristics, and prognosis to investigate the clinical usefulness of the two subtypes found by consensus clustering. Age, gender, T stage, N stage, follow-up period, and survival status were among the patient characteristics. Furthermore, the Kaplan-Meier curves created by the R packages “survival” and “survminer” were used to analyze OS differences across the three subtypes.

### 2.4. Correlations between Molecular Subtypes and TME in LC

Each patient's immunological and stromal scores were calculated using the ESTIMATE algorithm. Meanwhile, the CIBERSORT algorithm determined the scores of 22 human immune cell types for each LC sample [26]. In addition, the single-sample gene set enrichment analysis (ssGSEA) technique was utilized to estimate the amount of immune cell infiltration in LC patients' TME [27].

### 2.5. DEG Identification and Functional Annotation

DEGs between cuproptosis subtypes were found using the R program “limma,” and functional enrichment analysis of DEGs was done using the R tool “clusterprofiler.” The goal was to investigate the probable activities of cuproptosis pattern-associated DEGs further as well as to find related gene functions and enrichment pathways.

### 2.6. Construction of the Cuproptosis-Related Prognostic CRG_Score

To quantify cuproptosis patterns in individual tumors, cuproptosis scores were generated. First, DEG was subjected to univariate Cox regression analysis to discover relationships with OS in LC patients. Second, it was thoroughly examined by employing an unsupervised clustering technique based on the expression of prognostic CRG to categorize patients into several subtype groups (cuproptosis gene subtype A, cuproptosis gene subtype B, and cuproptosis gene subtype C). Finally, all LC patients were randomly assigned to one of two groups: a training group (*n* = 723) and a test group (*n* = 722), and a cuproptosis-related predictive CRG_score was calculated. Finally, we employed the “glmnet” R package which uses the Lasso Cox regression technique to reduce the danger of overfitting. Simultaneously, the change trajectory of each independent variable was examined, and the model was built using 10-fold cross validation. A prognostic CRG_score was calculated using a multivariate Cox analysis. CRG_score was computed as follows: (Expi^∗^coefi) = CRG_score. The Coefi and Expi values represent the risk coefficient and gene expression, respectively. A total of 723 patients in the training set and 722 patients in the testing set were split into low-risk (CRG_score median value) and high-risk (CRG_score > median value) groups and then submitted to the Kaplan-Meier survival analysis based on the median risk score. Meanwhile, all sets were separated into low- and high-risk groups, with each group subjected to the Kaplan-Meier survival analysis and the creation of receiver operating characteristic (ROC) curves. The “ggplot2” R software was then used to perform principal component analysis (PCA). Additional CCT6A, CD19, KYNU, SLC2A1, and ZBED1 protein levels of patients were individually detected by IHC in the HPA database.

### 2.7. Evaluation of Immune Status and Cancer Stem Cell (CSC) Index between the High- and Low-Risk Groups

We utilized CIBERSORT to count the number of 22 infiltrating immune cells in samples from low-risk and high-risk categories. Simultaneously, we investigated the relationship between 22 invading immune cells and prognosis-related genes in the CRG_score. We also utilized box plots to look at the differences in immune cell expression levels between low- and high-scoring groups. Furthermore, we investigated the link between the two risk categories and CSC.

### 2.8. Mutation and Drug Susceptibility Analysis

We utilized the R package “maftools” to build a mutation annotation format (MAF) from the TCGA database to identify somatic mutations in LC patients who were classified as high or low risk. In addition, for each LC patient in both groups, we generated tumor mutational burden (TMB) scores. To investigate the difference in the treatment impact of chemotherapeutic medications in the two groups of patients, we utilized the “pRRophetic” package to determine the half-inhibitory concentration (IC50) values of chemotherapeutic agents routinely used to treat LC.

### 2.9. Establishment and Validation of a Nomogram Scoring System

Clinical characteristics and risk scores were utilized to generate predicted nomograms based on the findings of separate prognostic studies using the ‘rms' software. Each variable of each patient may be matched with a score based on the patient's characteristics in the nomogram scoring system, and the scores of all variables of each sample can be summed to create a total score to estimate the patient's likelihood of survival [28]. Nomogram calibration plots are used to describe anticipated values between 3-, 5-, and 10-year survival events and actual observed results.

### 2.10. Statistical Analyses

All statistical analyses were performed using R version 4.2.0. Statistical significance was set at *P* < 0.05.

## 3. Results

**Figure 1:**
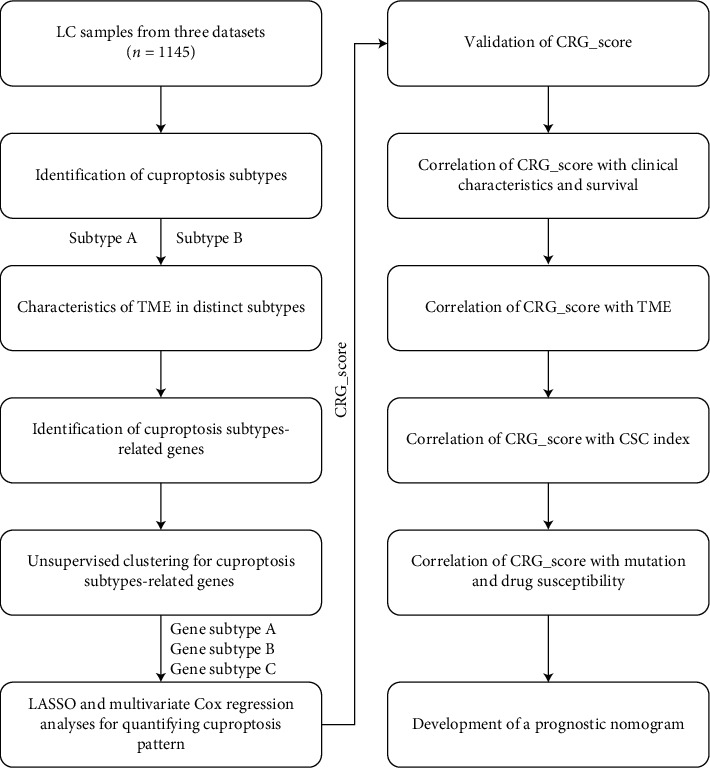
Flowchart of this study.

A detailed flowchart of this study is shown in Figure 1.

### 3.1. Genetic and Transcriptional Alterations of CRG in LC

**Figure 2:**
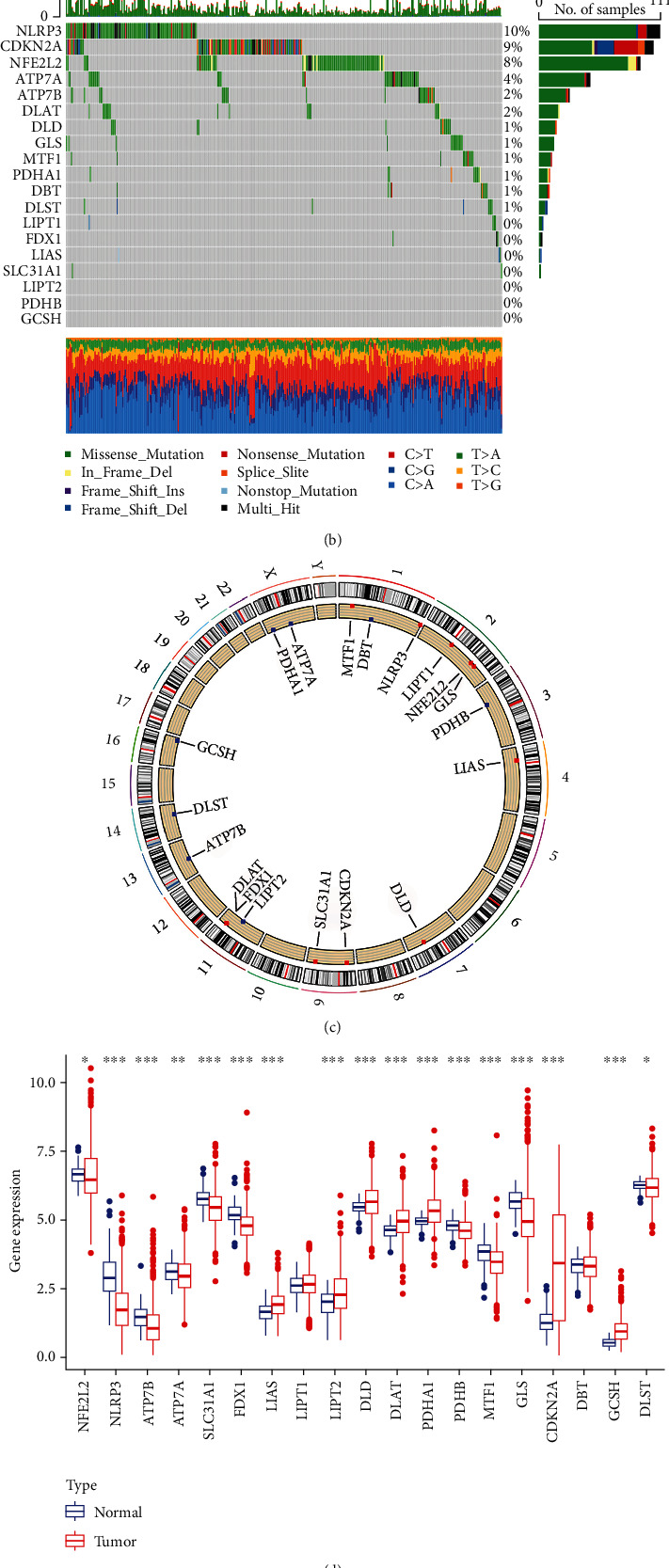
Genetic and transcriptional alterations of CRGs in LC.

This research includes all 19 CRG listed in the previous study. A comprehensive examination of the occurrence of somatic mutations in these 19 CRG found that 371 (31.96 percent) of the 1161 TCGA samples contained mutations in CRG (Figure 2(b)). NLRP3 exhibited the greatest mutation frequency (10%) followed by CDKN2A, whereas seven CRG (LIPT1, FDX1, LIAS, SLC31A1, LIPT2, PDHB, and GCSH) were mutation free.

Following that, we looked into somatic copy number alterations in these CRG and discovered that copy number changes were common in all 19 CRG. Copy number variations (CNVs) were frequently raised in NLRP3, NFE2L2, and LIPT2 but decreased in CDKN2A (Figure 2(e)).  Figure 2(c) depicts the chromosomal location of CNV changes in the CRG. We next analyzed the mRNA expression levels of LC patients' tumor tissues and normal tissues and discovered that most CRG expression levels were inversely linked with CNV changes. CNV-depleted CRG, such as CDKN2A, were expressed at greater levels in LC tumor tissue samples than in normal lung samples, but CNV-raised CRG, such as NLRP3 and NFE2L2, was considerably enhanced in LC tumor tissue samples (Figure 2(d)), indicating that CNVs may control CRG mRNA expression. Some CRG with CNV gain, such as LIPT2, showed increased mRNA expression; however, other CRG with high CNV gain or loss frequencies did not differ between tumor and normal samples. While CNVs may account for many of the observed variations in CRG expression, they are not the sole factors influencing mRNA expression. Other variables that can influence gene expression include DNA methylation and transcription factors. Our findings indicated substantial variations in the genetic landscape and expression levels of CRG between LC tissues and control samples, indicating that CRG may play a role in the carcinogenesis of LC patients.

### 3.2. Identification of Cuproptosis Subtypes in LC

We integrated and incorporated 1445 patients from four suitable LC cohorts (TCGA-LUAD, TCGA-LUSC, GSE41271, and GSE68465) into our study for a higher level of analysis to acquire a more complete knowledge of CRG expression patterns in carcinogenesis. The findings of univariate Cox regression and the Kaplan-Meier analysis indicated that nine CRG (MTF1, CDKN2A, ATP7A, DLD, LIPT1, SLC31A1, PDHB, DBT, and DLAT) had prognostic significance in LC patients (MTF1, CDKN2A, ATP7A, DLD, LIPT1, SLC31A1, PDHB, DBT, and DLAT) (Figure 2(a)). The cuproptosis network exhibited a synthesis of CRG interactions, regulator linkages, and their predictive relevance in LC patients (Figure 2(a)).

**Figure 3:**
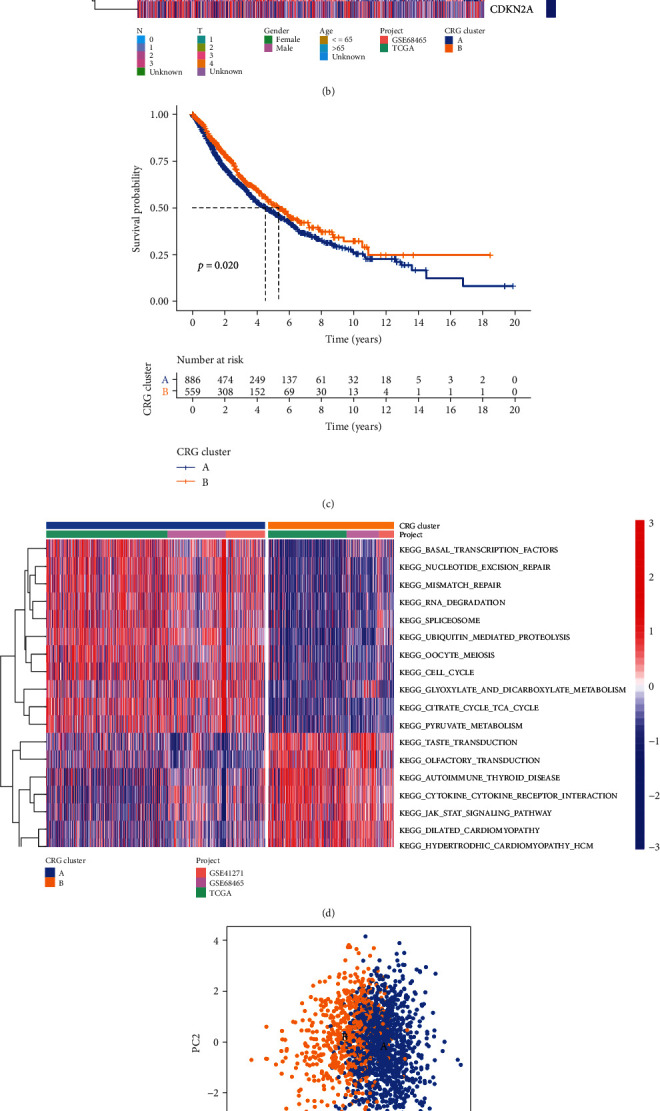
CRG subtypes and clinicopathological and biological characteristics of two distinct subtypes of samples divided by consistent clustering.

Based on the expression characteristics of the 19 CRG, we employed a consensus clustering technique to identify LC patients. Our findings indicated that setting the value of k to 2 was the optimal decision, and we divided the complete LC cohort into subtypes A (*n* = 1016) and B (*n* = 429) (Figure 3(a)), further examining the expression features of CRG in LC tumor tissues. PCA analysis revealed substantial heterogeneity in the transcriptional patterns of cuproptosis subtypes (Figure 3(e)). The Kaplan-Meier curves revealed that individuals with subtype B had a longer OS (log-rank test, *P* = 0.020; Figure 3(c)). A heatmap comparing the clinical and pathological aspects of distinct subtypes found substantial variations in CRG expression as well as clinical and pathological features (Figure 3(b)).

### 3.3. Characteristics of the TME in Distinct Subtypes

GSVA enrichment analysis showed that subtype A was significantly enriched in basal transcription factors, nucleotide excision repair, mismatch repair, RNA degeneration, cell cycle, and spliceosome pathways (Figure 3(d)). To investigate the role of CRG in the TME of LC, we assessed the correlations between the two subtypes and 22 human immune cell subsets of every LC sample using the CIBERSORT algorithm. We observed significant differences in the infiltration of most immune cells between the two subtypes. The infiltration levels of activated B cell, activated CD8 + T cell, activated dendritic cell, CD56 dim natural killer cell, eosinophil, immature B cell, MDSC, macrophage, mast cell, monocyte, natural killer T cell, natural killer cell, neutrophil, regulatory T cell, T follicular helper cell, type-1 T helper cell, and type-17 T helper cell were lower in the subtype A than those in the subtype B, while resting gamma delta T cell, immature dendritic cell, and type-2 T helper cell had significantly higher infiltration in subtype A compared to those in subtype B (Figure 3(f)).

### 3.4. Identification of Gene Subtypes Based on DEGs

**Figure 4:**
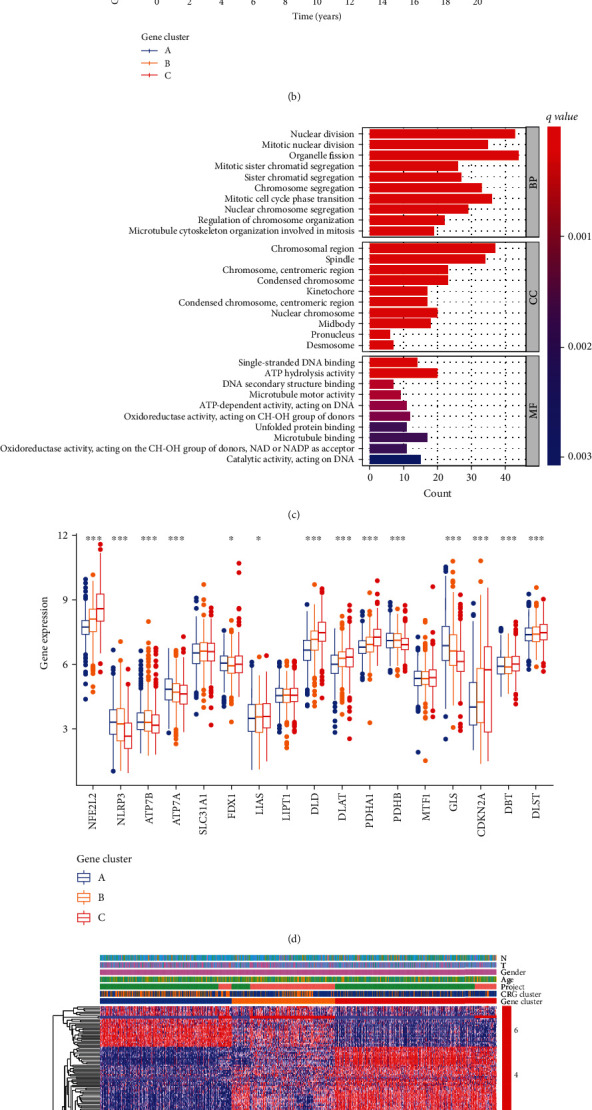
Identification of gene subtypes based on DEGs.

We discovered 377 DEGs linked with the cuproptosis subtype using the R package “limma” and performed a functional enrichment analysis (Figures 4(c)–4(f)). These DEGs associated with cuproptosis subtypes were considerably enriched in biological processes linked to cellular metabolism (Figure 4(c)). KEGG analysis revealed an enrichment of pathways associated with cell proliferation and cellular metabolism (Figure 4(f)), indicating that cuproptosis is important in TME cellular metabolism. These findings contribute to our understanding of the underlying biological activity of each cuproptosis type.

We used a consensus clustering technique to divide patients into three genomic categories based on prognostic genes to further confirm this regulatory mechanism (Figure 4(a)). Similarly, we created the Kaplan-Meier curves to assess survival disparities between different genomic subtypes, and the findings revealed that geneCluster C patients had the lowest OS (log-rank test, *P* = 0.001; Figure 4(b)). Furthermore, the data revealed that distinct genetic subtypes were linked to TN stage in LC patients (Figure 4(e)). Unsurprisingly, there were considerable disparities in CRG expression among the three genetic subtypes (Figure 4(d)).

### 3.5. The Development of a Nomogram to Predict Survival and the Construction and Validation of the Prognostic CRG_Score

**Figure 5:**
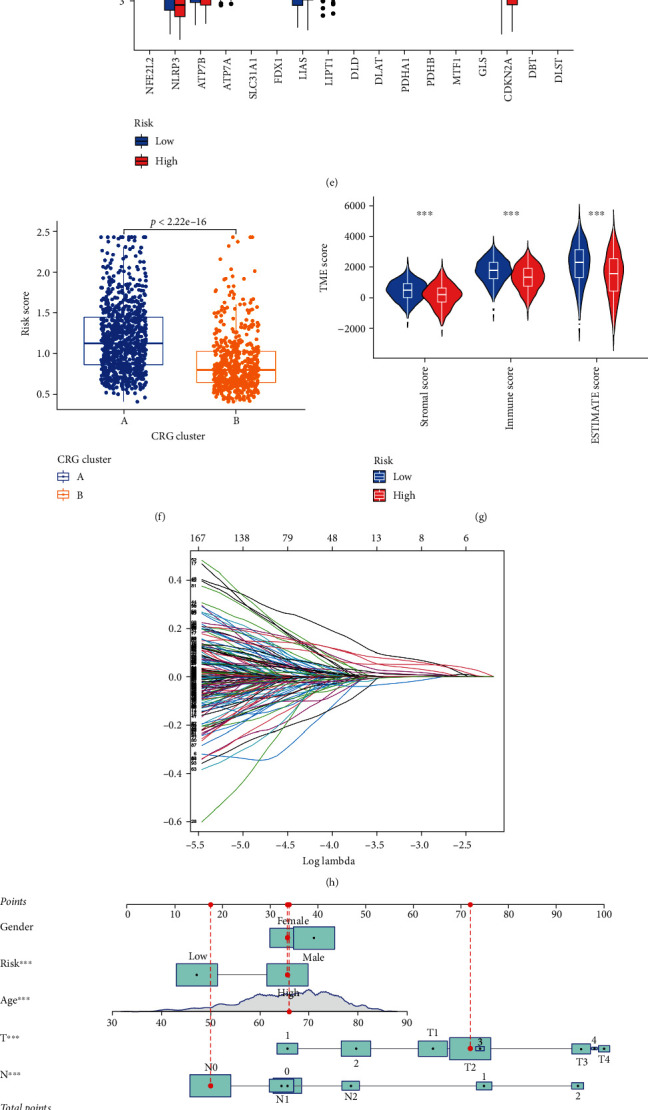
Correlations of tumor immune cell microenvironments and two LC subtypes.

The CRG_score was calculated using the subtype-related DEGs. First, we utilized R's “caret package” to randomly divide the patients into two groups: training (*n* = 723) and testing (*n* = 722). To choose the best prognostic characteristics, LASSO and multivariate Cox analyses were performed on 242 cuproptosis subtype-related prognostic DEGs. Following LASSO regression analysis, it was discovered that there were still 9 OS-related genes based on the least partial likelihood of deviation (Figures 5(d) and 5(h)). Based on the Akaike information criterion (AIC) values, we ran multivariate Cox regression analysis on the 9 OS-related genes, resulting in 5 (CCT6A, CD19, KYNU, SLC2A1, and ZBED1), including three high-risk genes (CCT6A, KYNU, and SLC2A1) and two low-risk genes (CD19 and ZBED1). The CRG_score was calculated using the findings of the multivariate Cox regression analysis as follows:

(1) $$\begin{array}{c} \text{Risk score}=\left(0.1622^{*}\text{expression of CCT}6\text{A}\right)+\left(-0.0780^{*}\text{expression of CD}19\right)+\left(0.1285^{*}\text{expression of KYNU}\right)+\left(0.1003^{*}\text{expression of SLC}2\text{A}1\right)+\left(0.1778^{*}\text{expression of ZBED}1\right). \end{array}$$

Figure 5(a) depicts the patient distribution in two cuproptosis subtypes, three gene subtypes, and two CRG_score groups. CRG_score differed significantly between cuproptosis gene subtypes. Subtype A had the lowest CRG_score, whereas subtype C had the highest (Figure 5(b)). We then split the 1445 LC patients into two groups: high risk and low risk. Patients with a CRG_score less than the median risk score were classified as low risk (*n* = 716), whereas those with a CRG_score more than the median risk score were classified as high risk (*n* = 729).  Figure 5(f) depicts the distribution of risk ratings for the two subgroups. In the HPA database, CCT6A, CD19, KYNU, SLC2A1, and ZBED1 protein levels of patients individually detected by IHC were presented in Supplementary Figure 2.

**Figure 6:**
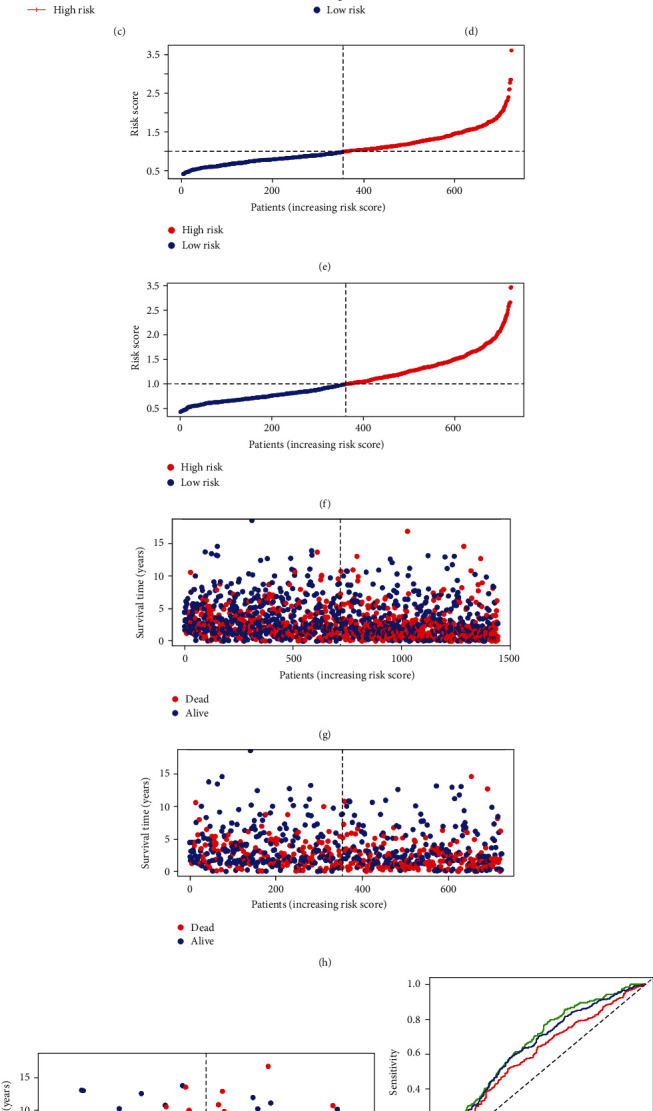
Construction of the CRG_score.

To further validate the CRG_score capacity to predict LC patient prognosis, we separated the training group (*n* = 723), testing group (*n* = 722), and all patients (*n* = 1445) into high-risk and low-risk groups based on CRG_score and generated their respective survival curves (Figure 6). Survival analysis revealed that the prognosis of the low-risk group was considerably better than that of the high-risk group in the training group (*n* = 722), testing group (*n* = 723), and all patients (*n* = 1445) (Figures 6(a)–6(c)).

We use a permuted dot plot to illustrate the CRG_score distribution and a scatter plot to show the patient's survival status (Figures 6(d)–6(i)). Meanwhile, our investigation of 1-, 3-, and 5-year prognostic prediction classification efficiency revealed that CRG_score retained a very high AUC value (Figures 6(j)–6(l)), showing that the CRG_score had an exceptional capacity to predict LC patient survival.

Clinically, CRG_score alone cannot predict OS in LC patients. We created a nomogram including CRG_score and clinical and pathological characteristics to predict 1-, 3-, and 5-year OS rates to make clinical use of CRG_score easier (Figure 5(i)). Predictors included the patient's CRG_score as well as age, gender, and TN stage. Supplementary Figure 3 showed the K-M of lung cancer patients with clinical characteristics such as age (a), gender (b), T (c), and N (d) with prognosis. The calibration plots reveal that our built nomograms can predict survival well (Figure 5(c)).

### 3.6. Evaluation of TME between the High- and Low-Risk Groups

We used the CIBERSORT method to examine the relationship between CRG_score and immune cell abundance. Furthermore, our findings revealed that a low CRG_score was highly related to a low immunological score, stromal score, and projected score (Figure 5(g)). We also looked at the link between five genes in the suggested model and the number of immune cells. We discovered that the five genes were highly connected with the majority of immune cells (Figure 5(k)).  Figure 5(e) showed that 14 genes were differentially expressed in the two groups. The CRG_score was associated with neutrophils, activated mast cells, resting mast cells, resting dendritic cells, M1 macrophages, M0 macrophages, monocytes, memory B cells, plasma cells, follicular helper T cells, activated memory CD4 + T cells, resting memory CD4 + T cells, Tregs, resting NK cells, and activated NK cells, as shown in the scatter diagrams (Supplementary Figure 1).

### 3.7. Relationship of CRG_Score with CSC Index

Furthermore, we investigated the possible relationship between CRG_score and CSC in LC patients. Our findings revealed a significant relationship between CRG_score and CSC index (*R* = 0.19), and this linear analysis revealed that LC cells with higher CRG_score had less cellular differentiation and more prominent stem cell traits (Figure 5(j)).

### 3.8. Mutation and Drug Susceptibility Analysis

**Figure 7:**
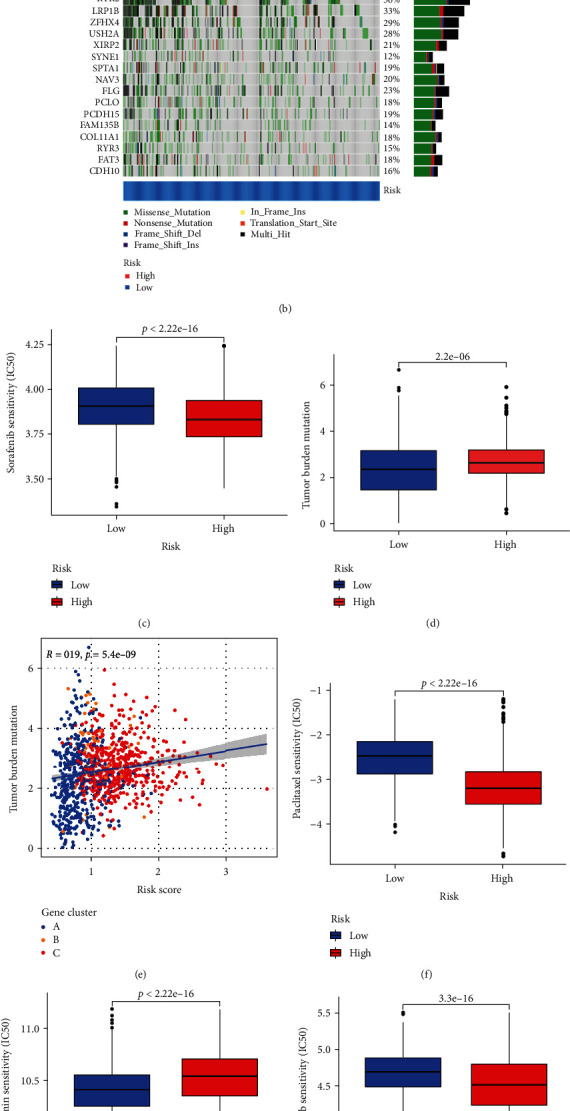
Comprehensive analysis of the CRG_score in LC.

TMB was higher in the high group than in the low group, according to our analysis of mutational data from the TCGA LUAD/LUSC cohort (Figure 7(d)), suggesting that immunotherapy may help the high-risk group. We then looked at how the distribution of somatic mutations differed between the two CRG_score groups. TP53, TTN, MUC16, CSMD3, RYR2, LRP1B, ZFHX4, USH2A, and XIRP2 were the top 10 mutant genes in both the high-risk and low-risk groups (Figures 7(a) and 7(b)). When compared to patients with low CRG_score, individuals with high CRG_score showed considerably greater mutation rates in these genes (Figures 7(a) and 7(b)). The Spearman correlation analysis demonstrated that the CRG_score was positively associated with the TMB (*P* < 0.001; Figure 7(e)). We next chose other commonly used chemotherapeutic medicines to test the sensitivity of low-risk and high-risk patients to these medications. Patients with a low CRG_score exhibited significantly higher IC50 values for chemotherapeutic medicines such as erlotinib, gefitinib, sorafenib, and paclitaxel. Interestingly, in addition to chemotherapeutic medications, we discovered that CRG_score was linked with metformin IC50 value, and metformin IC50 value was considerably greater in patients with high CRG_score. Taken together, these findings indicate that CRG are linked to drug sensitivity (Figures 7(c) and 7(f)–7(i)).

## 4. Discussion

Lots of research had revealed that copper is intimately linked to the incidence and development of different malignancies and that cytotoxicity generated by copper ion imbalance is linked to cancer cell proliferation and dissemination [29]. Further research into the mechanism of enhanced intracellular toxicity produced by copper ion imbalance might provide fresh insight into the efficient destruction of cancer cells in immunotherapy [30]. Tsvetkov et al., Tang et al., and Wang et al. found an altogether new process of cell death dubbed cuproptosis in a newly published study that is completely distinct from recognized types of cell death (thermal apoptosis, apoptosis, ferroptosis, and necroptosis) [12, 31, 32]. Currently, no research has been conducted to determine the function of copper death in innate immunity and antitumor effects. Meanwhile, the combined action of CRG has not completely revealed the overall consequences and TME infiltration features. Given this, the development of copper ion treatment regimens targeting cancers with this metabolic profile appears promising. Finding biomarkers that can identify cuproptosis in human tumor tissue is thus a novel method of cancer treatment with far-reaching implications. The findings of this study show that CRG is altered at the transcriptional and genomic levels in LC. We detected two different molecular subtypes and evaluated clinicopathological abnormalities and OS differences between the two subtypes as well as substantial variations in TME features. Significant immunological activity is another feature of the LC subtype. Furthermore, based on the DEG between the two copper death isoforms, we found three genotypes. We developed a reliable and effective prognostic CRG_score and proved its predictive ability. Then, our research found that individuals with low-risk and high-risk CRG_score had substantial differences in clinicopathological characteristics, prognosis, mutations, TME, CSC index, and medication sensitivity. Finally, by combining the clinical and pathological characteristics of the CRG_score and LC samples, we created a nomogram that is easy to utilize in clinical settings, significantly boosting the performance and clinical value of the CRG_score. Our work is the first to use CRG to predict LC prognosis, which aids in understanding the molecular mechanism of CRC and gives new ideas for targeted therapy. The predictive model we developed can also be utilized clinically to stratify LC patients' prognosis.

Despite recent breakthroughs in immunotherapy, the prognosis of LC patients remains variable, emphasizing the importance of TME in the development and progression of LC tumors. The TME is made up of immune cells (granulocytes, lymphocytes, and macrophages) and is engaged in a variety of immunological responses and activities as well as having a substantial influence on tumor growth, progression, and treatment resistance [33–35]. We discovered that the features of TME differed considerably across the two molecular subtypes and distinct CRG_score in this investigation. This data implies that CRG plays an important role in the evolution of LC. Subtype B, which had a better prognosis and a lower CRG_score, had more activated B cells and CD8+ T cells infiltrate, suggesting that they play an essential role in the development of LC. Tregs' primary function is to inhibit the anticancer immune response, which is linked to a poor prognosis. This is consistent with our observation that patients with subtype B and a low CRG_score had more Tregs in the TME than patients with a low CRG_score. Recent research has demonstrated that B cells have a role in the immunological response [36, 37]. We also found significant variations in memory B cell infiltration between the two subtypes and the CRG_score group in our investigation.

The CRG_score developed in this work was made up of five cuproptosis-related genes (CCT6A, CD19, KYNU, SLC2A1, and ZBED1). Chaperonin-containing tailless complex polypeptide 1 (CCT) is a protein complex that folds actin and tubulin. It is made up of eight different subunits (designated CCT1 to CCT8) [38]. CCT6A was discovered to be an inhibitor and direct binding protein of SMAD2, and it was shown that CCT6A may block the function of SMAD2 in NSCLC cells and enhance cancer cell metastasis [39]. CD19+ B cells are the second most common immune cell type in NSCLC tumors (16%) [40]. In addition, CD19 is the main target molecule of chimeric antigen receptor (CAR) T cells [41]. Studies have shown that absolute CD19+ counts in NSCLC patients are significantly lower than in age-matched controls [42]. Kynureninase (KYNU), a tryptophan metabolism hydrolase, contributes to the production of NAD+cofactors via the kynurenine pathway, and KYNU is implicated in the formation and spread of breast cancers [43]. The research of Fahrmann et al. discovered that NRF2 can inhibit tumor immunosuppression in LC by upregulating KYNU. Furthermore, higher KYNU is linked to immunosuppression and worse survival [44]. Many studies have discovered a link between SLC2A1 and the clinical features and prognosis of LC patients. The majority of research has shown that SLC2A1 downregulation is related to a better prognosis for LC patients [45–47]. The zinc-finger, BED-type (ZBED) gene family is a closely related genome that encodes regulatory proteins to help regulate various functions, widespread in vertebrate tissues [48]. Studies have shown that ZBED1 is highly expressed in gastric cancer, and higher ZBED1 levels predict poor outcomes. [49]. However, no research has been conducted to investigate the association between ZBED1 and LC as well as its relationship with LC prognosis and clinicopathological features. According to our findings, low ZBED1 expression is related to a decreased risk of LC. CCT6A, KYNU, and SLC2A1 were shown to be high-risk genes for LC prognosis, while CD19 and ZBED1 were found to be low-risk genes. This demonstrates the great accuracy and clinical applicability of our scoring method, which is consistent with the previous findings.

There are several limitations to this study. First, all analyses were conducted using data from public sources, and large-scale prospective studies as well as further in vivo and in vitro experimental research are needed to corroborate our findings. Second, although the predictive effect of the nomogram model developed in this study was more favorable (Figure 5(c)). However, as shown in Supplementary Figure 3 and Figure 5(i), the risk score of lung cancer patient in this study had a smaller score weight in the nomogram compared with the classical TNM staging, which may lead to a smaller predictive power of the risk score in this study, and the inclusion of more patients may be needed in the future to assess the weight of the risk score in the nomogram. Nevertheless, the risk score established in this study remains an important predictor of patient prognosis. Furthermore, data on several essential clinical variables, including surgery, radiation, and chemotherapy, are not captured in most datasets, which may have an impact on our study of prognostic factors and the development of prognostic models. Prospective studies are also required to improve these findings.

## 5. Conclusions

Our thorough analysis of CRG showed a complex regulatory system by which they influence the tumor-immune-stromal microenvironment, clinical and pathological characteristics, and prognosis. We also investigated CRG's therapeutic potential in targeted treatment and immunotherapy. These findings emphasize CRG's critical therapeutic relevance and offer fresh ideas for steering individualized immunotherapy efforts for LC patients.

## Data Availability

TCGA dataset was downloaded from Xena (https://xenabrowser.net/datapages/, TCGA-LUAD, TCGA-LUSC). Multiomics data was downloaded from data portal (https://portal.gdc.cancer.gov/, TCGA-LUAD, TCGA-LUSC). Other transcriptome datasets were downloaded from Gene Expression Omnibus (http://www.ncbi.nlm.nih.gov/geo/). Immune-related genes were obtained from InnateDB (https://www.innatedb.com) and Immport (https://www.immport.org/). Drug sensitivity data was obtained from PRISM (https://depmap.org/portal/prism/) and CTRP (https://portals.broadinstitute.org/ctrp).

## Supplementary Materials

Supplementary materials Supplementary Figure 1: The CRG_score was associated with immune cells. Supplementary Figure 2: CCT6A, CD19, KYNU, SLC2A1, and ZBED1 protein levels of patients individually detected by IHC. Supplementary Figure 3: The K-M of lung cancer patients with clinical characteristics.
