# Supplementary material for: Identification of Cuproptosis-Related Subtypes in Lung Cancer, Characterization of Tumor Microenvironment Infiltration, and Establishment of a Prognostic Model
Source: Biomed Res Int. 2022 Dec 21;2022:7406636. doi: 10.1155/2022/7406636 (PMC9797313; doi:10.1155/2022/7406636)

**Supplementary Figure.1 The CRG_score was associated with immune cells**

**
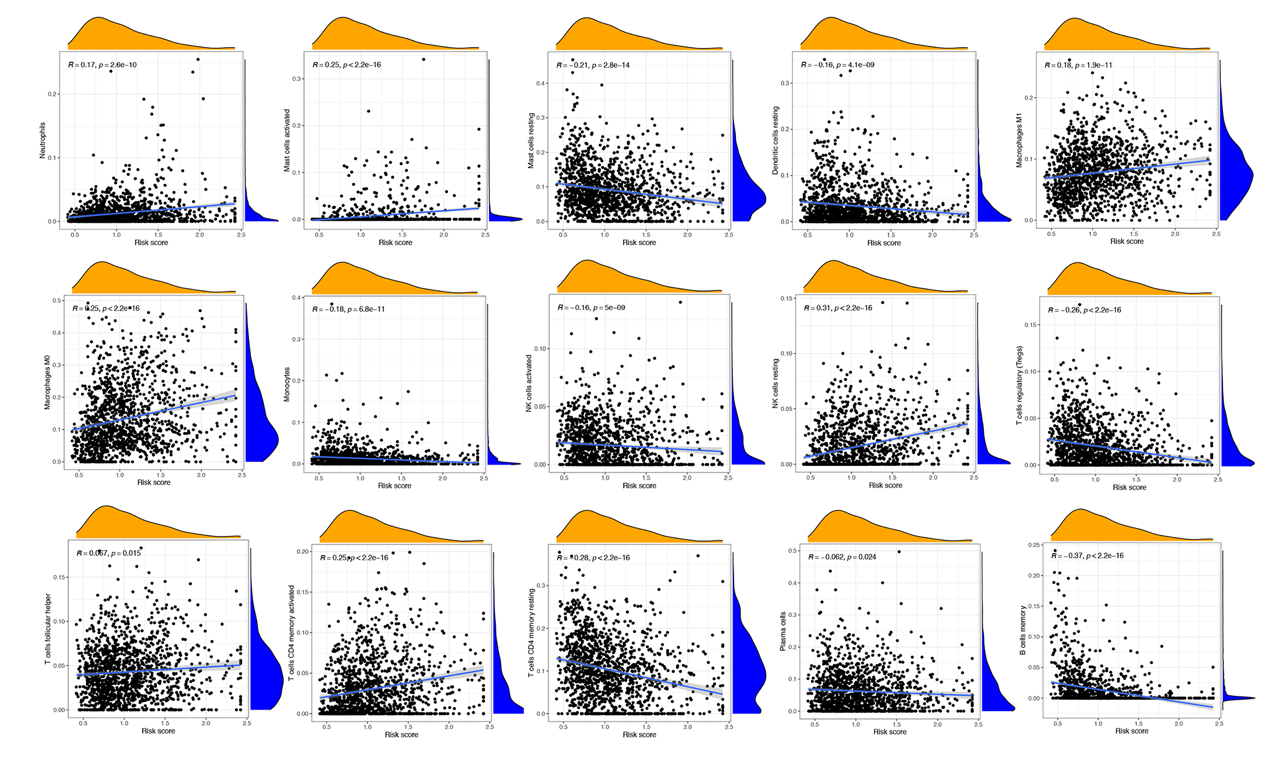
**

**Supplementary Figure.2 CCT6A, CD19, KYNU, SLC2A1 and ZBED1 protein levels of patients individual detected by IHC.**

**
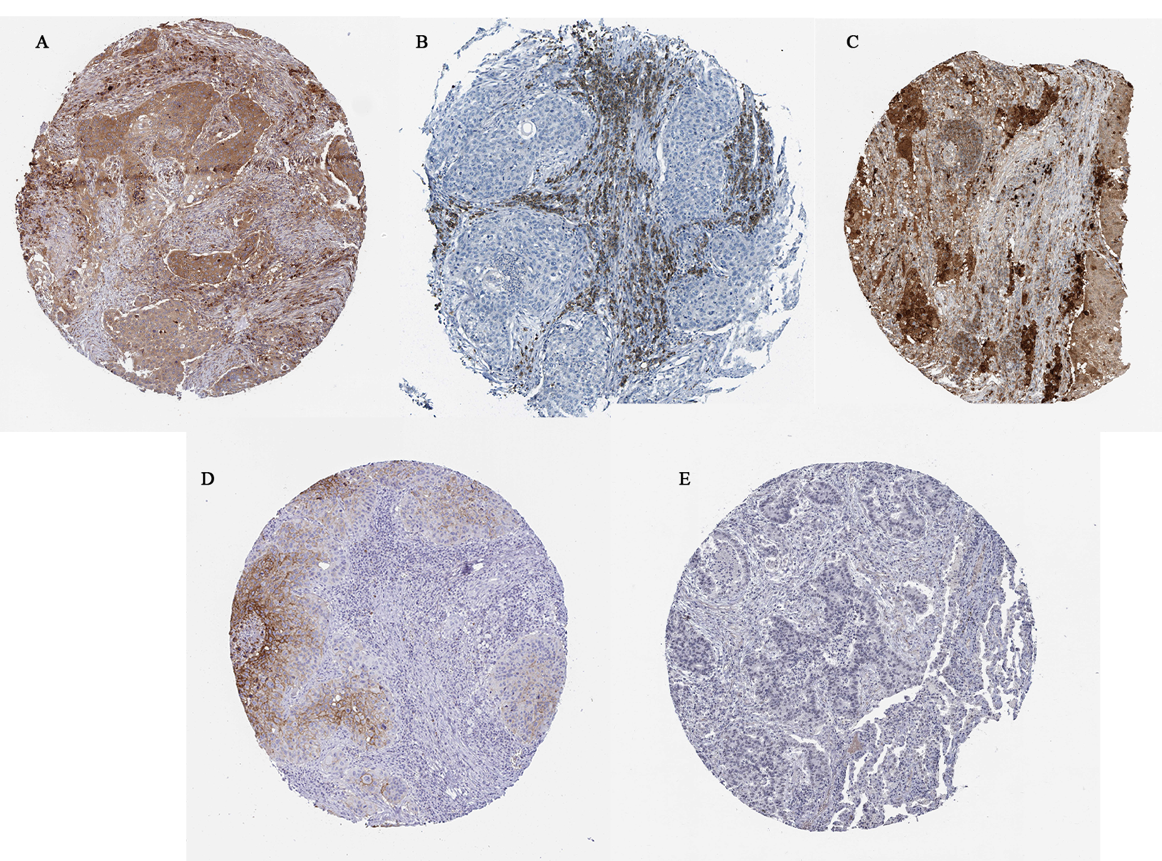
**

**Supplementary Figure.3 The K-M of lung cancer patients with clinical characteristics.**


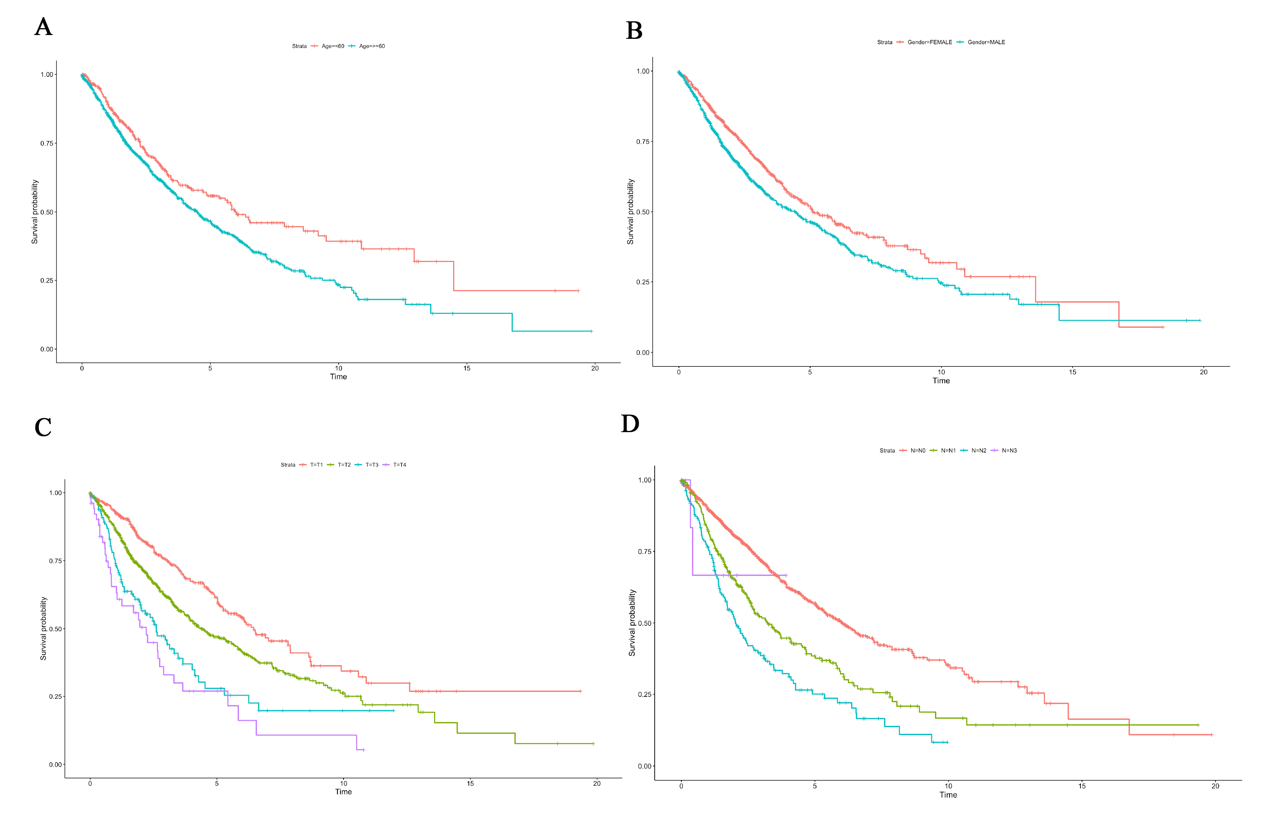

Supplement: Supplementary materials — Supplementary Figure 1: The CRG_score was associated with immune cells. Supplementary Figure 2: CCT6A, CD19, KYNU, SLC2A1, and ZBED1 protein levels of patients individually detected by IHC. Supplementary Figure 3: The K-M of lung cancer patients with clinical characteristics. [file 7406636.f1.docx]
